# Supplementary material for: Possible Metastatic Stage-Dependent ILC2 Activation Induces Differential Functions of MDSCs through IL-13/IL-13Rα1 Signaling during the Progression of Breast Cancer Lung Metastasis
Source: Cancers (Basel). 2022 Jul 4;14(13):3267. doi: 10.3390/cancers14133267 (PMC9265472; doi:10.3390/cancers14133267)
Supplement: Supplementary file 1 [file cancers-14-03267-s001.zip › cancers-1716724-supplementary.pdf]

Supplemental Figure S1

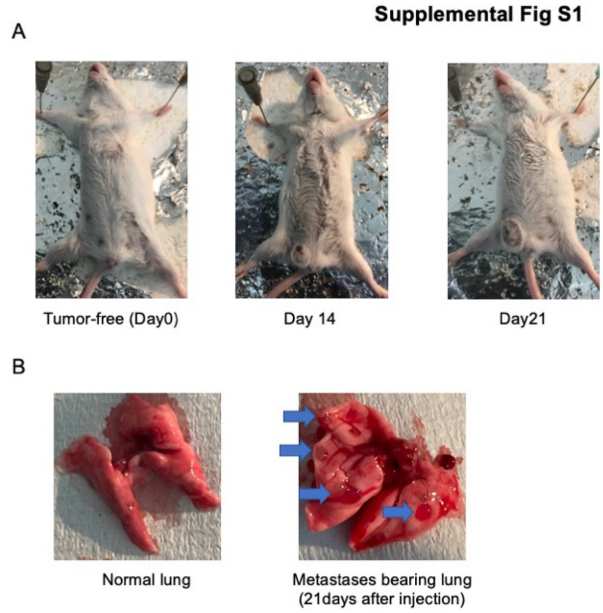

(A) Representative photograph of 4T1/LM4 tumor-bearing mice. The primary tumor volume constantly increased in a time-dependent manner (Left: tumor free; Center: 14 days after tumor-injection; Right: 21 days after tumor-injection).
(B) Representative photographs of the lungs of tumor-free mice and 4T1/LM4 tumor-bearing mice (21 days after tumor-injection) are shown. Arrows indicate the metastatic foci of metastases-bearing lungs.

Supplemental Figure S2

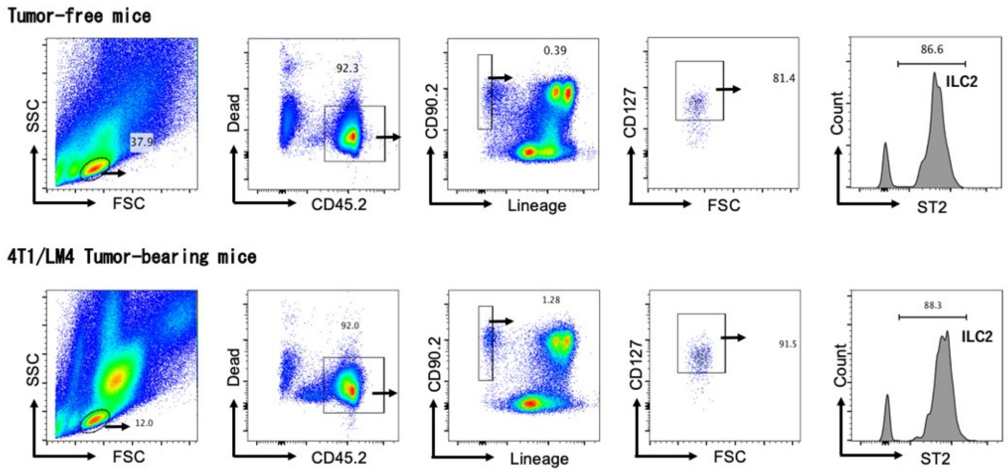

Representative flow cytometry dot-plots of ILC2 gating strategy for the lungs of tumor-free and 4T1/LM4 tumor-bearing mice are shown. Leukocytes were isolated and stained with anti-mouse CD45.2, CD90.2, CD127, ST2, Zombie Aqua antibodies and a lineage cocktail, and were then subjected to flow cytometry to evaluate their expression. Data are representative of at least three independent experiments that show similar results.

Supplemental Figure S3

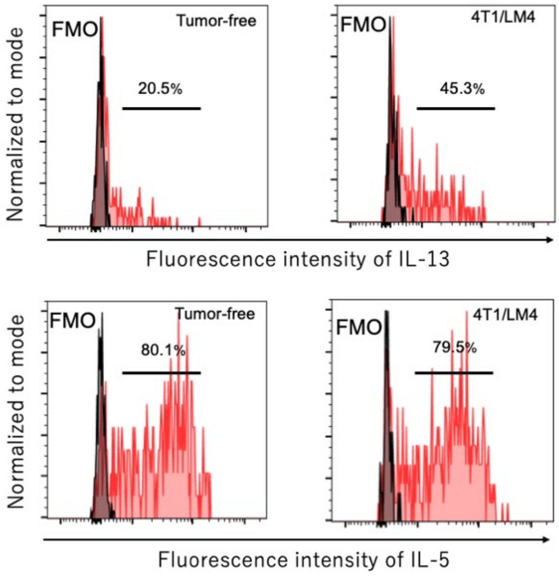

Representative flow cytometry histograms of IL-13 and IL-5-producing ILC2s in the lungs from tumor-free and 4T1/LM4 tumor-bearing mice. Positive signals were identified by comparing staining with a fluorescence minus one control sample. FMO indicates fluorescence minus one control.

Supplemental Figure S4

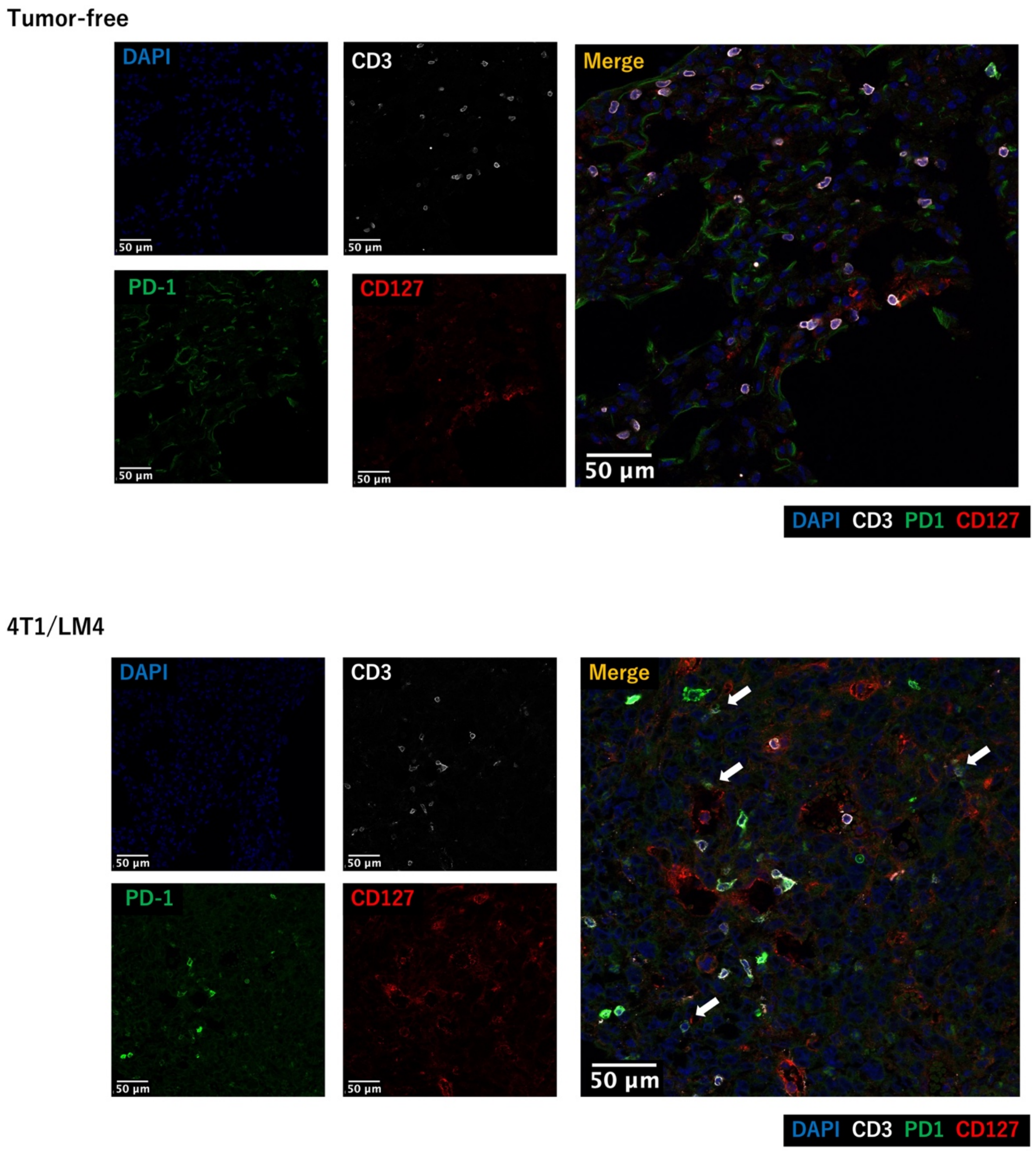

Immunofluorescent staining of CD3 (white), CD127 (Red) and PD-1 (Green) in mouse lung tissues. $CD3^{-}CD127^{+}PD\text{-}1^{+}$cells, which indicated $PD\text{-}1^{+}$ ILCs (shown by arrows), were increased in the lungs of 4T1/LM4 tumor-bearing mice compared to tumor-free mice. In addition, $CD3^{+}PD\text{-}1^{+}$ cells, which indicated $PD\text{-}1^{+}$ T cells, were also more abundant in the lungs of tumor-bearing mice than in tumor-free mice. Lungs of tumor-free mice and 4T1/LM4 tumor-bearing mice were fixed in 4% paraformaldehyde for 4hr prior to being embedded in OCT freezing media (Sakura Finetek, Japan). 8μm sections were cut on a CM1850 cryostat (Leica Microsystems) and affixed to glass microscope

slides (Matsunami, Japan). Frozen sections were blocked with PBS including 0.1% Triton-X100 (Sigma), 1% BSA and 10% FBS at room temperature for 1hr. After washing with PBS three times, the section was stained with rabbit anti-mouse PD-1 mAb (D7D5W, Cell Signaling Technology), rat anti-mouse CD127 mAb (A7R34, Biolegend), and Syrian hamster anti-mouse CD3 mAb (500A2, Biolegend) at 4℃ for overnight. To detect each signal, donkey anti-rabbit Alexa 488 (Invitrogen), goat anti-rat Alexa 555 (Invitrogen), goat anti-Syrian hamster Alexa 647 (Jackson ImmunoResearch Laboratories) were used. DAPI stained slides were examined with a Leica TCS SP8 confocal microscope (Leica Microsystems GmbH, Mannheim, Germany).

Supplemental Figure S5

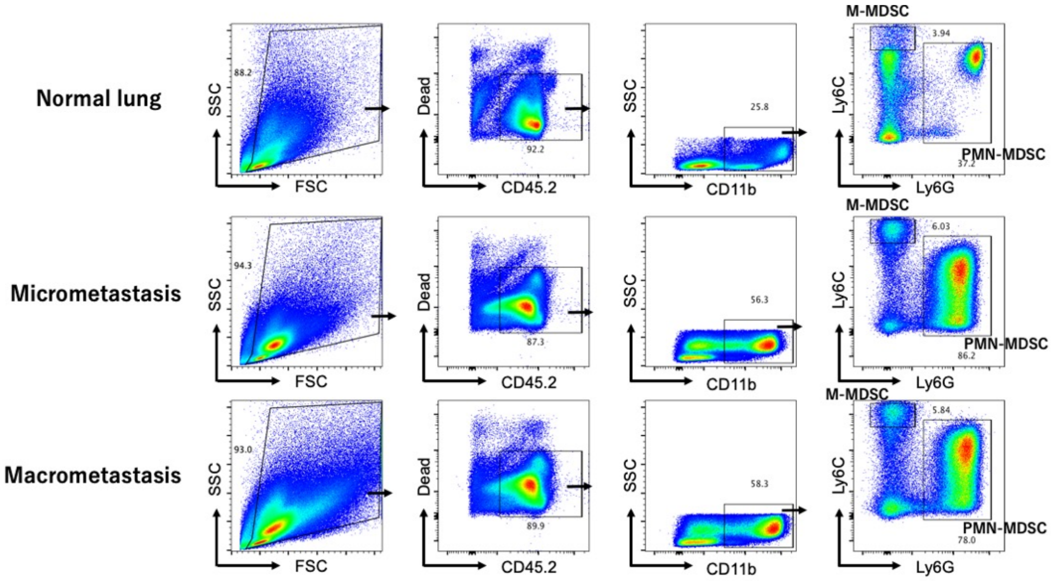

Representative flow cytometry dot-plots gating strategy for PMN-MDSCs and M-MDSCs from the normal lungs of tumor-free mice, as well as from the micro- and macrometastatic regions of 4T1/LM4 tumor-bearing mice. Single-cell suspensions were stained with antibodies to mouse CD45.2, CD11b, Ly6G, Ly6C and Zombie Aqua and subjected to flow cytometry to evaluate their expression. Data are representative of at least two independent experiments that show similar results.

Supplemental Figure S6

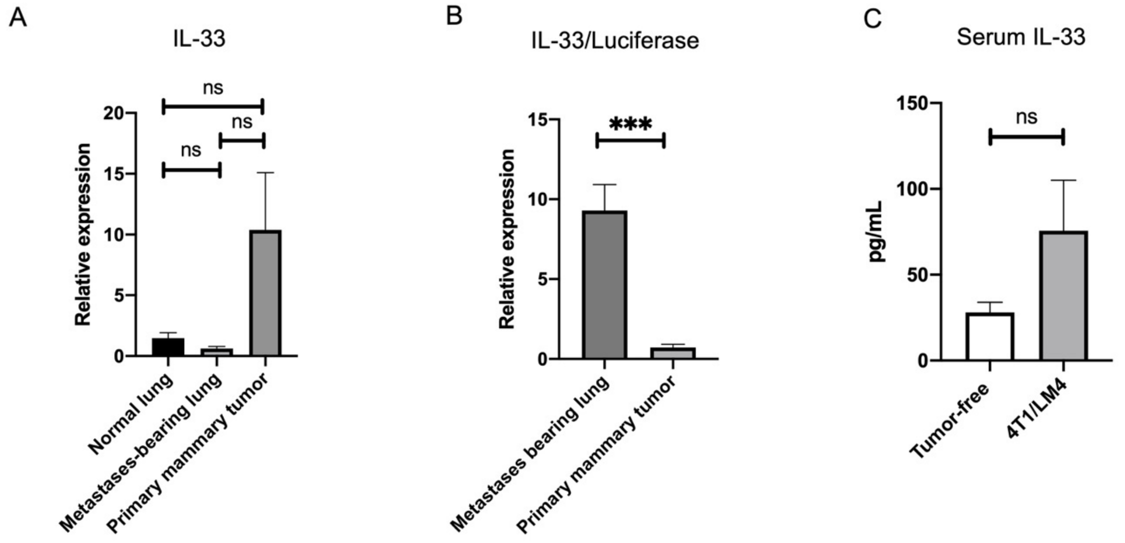

(A) RT-qPCR analysis for gene expression of IL-33. Total RNA was isolated from the lungs of tumor-free mice, the metastases-bearing lungs and primary mammary tumors and analyzed for mRNA expression of IL-33 (Normal lung: n=4, Metastases-bearing lung: n=5, Primary mammary tumor: n=7). *Actb* mRNA served as an endogenous control to normalize mRNA levels using the $2^{-\Delta\Delta CT}$ method. Statistical significance was obtained by ordinary one-way ANOVA; this was followed by a Tukey's multiple comparisons test for multiple groups. ns indicates not significant. Data represent the mean ± SEM.

(B) RT-qPCR analysis for gene expression of IL-33 using luciferase as a loading control. Total RNA was isolated from the metastases-bearing lungs and primary mammary tumors and analyzed for mRNA expression of IL-33 (Metastases-bearing lung: n=3, Primary mammary tumor: n=5). 4T1/LM4 specific luciferase served as a loading control to normalize mRNA levels using the $2^{-\Delta\Delta CT}$ method. Data represent the mean ± SEM. The data from metastases-bearing lungs and primary mammary tumors were evaluated using the unpaired t-test. ***P < 0.001.

(C) Measurement of serum IL-33. The mouse blood was drawn by cardiac puncture and collected in heparinized tubes (Tumor-free mouse: n=6, 4T1/LM4 mouse: n=8). The concentrations of IL-33 in the mice serum samples were measured by enzyme-linked immunosorbent assay (ELISA). Data represent the mean ± SEM. The data from tumor-free and 4T1/LM4 mice were evaluated using the unpaired t-test.

Supplemental Figure S7

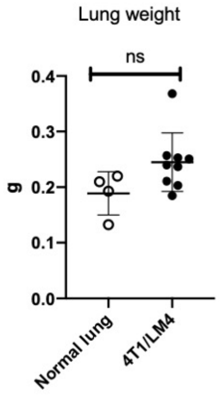

Lung weights of tumor-free and 4T1/LM4 tumor-bearing mice were compared (Control: n=4, 4T1/LM4: n=9). Data represent the mean ± SEM. The data from control and 4T1/LM4 tumor-bearing mice were evaluated using the unpaired t-test. Data represent the mean ± SEM. ns indicates not significant.
